# General *versus* spinal anesthesia in percutaneous nephrolithotomy: A systematic review and meta-analysis

**DOI:** 10.12688/f1000research.124704.1

**Published:** 2023-03-14

**Authors:** Rinaldo Indra Rachman, Ponco Birowo, Ghifari Nurullah, Prof. Sung Yong Cho, Widi Atmoko, Indah Suci Widyahening, Nur Rasyid

**Affiliations:** 1 Department of Urology, Faculty of Medicine. Universitas Indonesia. Cipto Mangunkusumo Hospital, Jalan Diponegoro No. 71, Jakarta, 10430, Indonesia; 2 Department of Urology, Seoul National University College of Medicine, Seoul National University Hospital, Seoul, 101 Daehak-Ro Jongno-Gu, South Korea; 3 Department of Community Medicine, Faculty of Medicine. Universitas Indonesia, Jalan Pegangsaan Timur no 16, Jakarta, 10430, Indonesia

**Keywords:** PCNL, Spinal Anesthesia, General Anesthesia, Complication, Stone-free Rate

## Abstract

**Background**: Percutaneous nephrolithotomy (PCNL) is the preferred treatment for the removal of large kidney stones, sized >20 mm. However, there is still an ongoing debate concerning the best anesthesia for PCNL. This study aimed to compare the efficacy and safety between general and spinal anesthesia for PCNL.

**Methods**: A systematic review and meta-analysis study. A systematic, electronic literature search was performed in several databases, including PubMed, Scopus, and Google Scholar until July 1 ^st^, 2022. The quality of the articles was examined using Crombie's Items (for non-randomized controlled trials (RCTs)) and Jadad Scale (for RCTs). The outcomes assessed were operation time, fluoroscopy time, length of stay, stone-free rate, overall complication rate, specific postoperative complications, cost, pain score, and postoperative analgesic requirement. The article selection was reported based on the Preferred Reporting Items for Systematic Reviews and Meta-Analysis (PRISMA) guidelines. We assessed four RCTs and eight retrospective studies. Meta-analysis of selected studies was performed using the Review Manager 5.3.

**Results:** General anesthesia resulted in fewer Clavien–Dindo grade II (OR: 0.68; 95% CI: 0.49 – 0.94; p=0.02), major complications (OR: 0.65; 95% CI: 0.45 – 0.94; p=0.02, and lower transfusion rates (OR: 0.70; 95% CI: 0.53 – 0.94; p=0.02). Whereas spinal anesthesia resulted in faster operation time (Mean Difference: -12.98; 95% CI: -20.56 – -5.41; p<0.001, fluoroscopy time (MD: -26.15; 95% CI: -42.79 – -9.50; p=0.002), reduced length of stay (MD: -0.47; 95% CI: -0.75 – 0.20; p<0.001), and lower postoperative analgesic requirement and cost. No significant difference in stone-free rate (OR: 1.08; 95% CI: 0.92 – 1.26; p=0.37). PCNL performed using either general anesthesia or spinal anesthesia is equally safe and effective.

**Conclusions:** Each method of anesthesia has its own advantages and disadvantages. The final choice between general and spinal anesthesia should be based on the patient's condition and surgical team preference.

## Introduction

Nephrolithiasis remains a common health problem around the globe. Its prevalence is 7–13% in North America, 5–9% in Europe, and 1–5% in Asia. According to the European Association of Urology (EAU) and American Urological Association (AUA), percutaneous nephrolithotomy (PCNL) is the first line treatment modality for renal calculi sized >20 mm. ^1^^,^^2^ PCNL is also useful for treating multiple stones, staghorn stones, and stones that are resistant to extracorporeal shockwave lithotripsy. ^1^^,^^2^ There are variations to PCNL, including position, imaging modality, dilation method, and anesthesia method. ^3^^,^^4^

There is conflicting evidence between the appropriate use of general anesthesia (GA) *versus* spinal anesthesia (SA) for PCNL. GA was associated with a longer duration of surgery and postoperative length of hospital stay in most studies. ^3^^,^^5^^–^^8^ GA allows greater flexibility for the anesthesiologist to extend the duration of anesthesia, whereas in SA, this would be more problematic. SA is associated with better postoperative pain control, thereby reducing the need for analgesic drugs. ^5^^,^^8^ Some studies have also shown that GA costs more than SA and has a higher rate of complications. The complications usually occur when the patient's position is altered from supine to prone. These complications include brachial plexus injury, spinal cord injury, and lung injury. ^8^^,^^9^

The aim of this study was to evaluate the safety and efficacy profile, in terms of stone free rate, of GA and SA in PCNL.

## Methods

### Description of conditions and interventions

This is a systematic review and meta-analysis study evaluating the efficacy and safety of SA compared to GA in PCNL. We included studies that involved patients with renal calculi sized >20 mm who underwent PCNL. The intervention group included patients who were administered SA, whereas the control group were administered GA. The safety profile was determined using the complication rate (classified by the Clavien–Dindo scoring system) and degree of specific complications (headache, urinary tract infection (UTI), urosepsis, and transfusion rate). The efficacy profile was determined using values of stone free rate, operation duration, and length of stay. Any studies that included patients with any renal anomaly, such as horseshoe kidney, malrotated kidney, or ectopic kidney were excluded. Furthermore, this study also excluded studies involving patients with contraindications for SA and GA, such as spinal deformity, severe cardiac and respiratory failure, or severe renal failure.

### Database search and literature screening

A systematic search of the literature was performed until July 1 ^st^, 2022, using PubMed (RRID:SCR_004846), Scopus (RRID:SCR_022559), and Google Scholar (RRID:SCR_008878) databases. The keywords used were “Spinal, General, Percutaneous Nephrolithotomy, PCNL, PNL” in PubMed, “spinal, general, anesthesia, percutaneous nephrolithotomy” in Scopus, and “spinal, spine, general, PCNL, PNL, percutaneous, nephrolithotomy” in Google Scholar. All keywords were combined using the Boolean logic. The articles identified were then screened for duplicates, which were then removed. The article selection was reported based on the Preferred Reporting Items for Systematic Reviews and Meta-Analysis (PRISMA) guidelines. ^29^

### Study selection

Two reviewers (RIR and PB) examined the articles independently. In case of any disagreement, a discussion was conducted to resolve the matter. The articles were screened for their relevance through the titles and abstracts. The inclusion criteria were comparative studies or randomized controlled trials (RCTs) concerning the use of SA and GA in PCNL. All included articles were written in English. The exclusion criteria were non-comparative studies; studies that combined SA with another method of anesthesia, such as epidural anesthesia; studies with irrelevant outcomes; and studies that included patients with renal anomalies, such as malrotated kidney, horseshoe kidney, or ectopic kidney. The quality of the articles was examined using the Crombie's Items scale (for non-RCTs) and Jadad Scale (for RCTs). ^10^^,^^11^

### Data extraction

The process of data extraction of the articles was independently conducted by two reviewers (GN and PB), and any disagreement was resolved by consensus. The variables extracted from the articles were article title, author's name, year of publication, stone free rate, length of stay, operation duration, fluoroscopy time, complication rate (classified using Clavien–Dindo scores), and specific complication rate (headache, UTI, urosepsis, and transfusion rate). Major complication rate was defined by a Clavien–Dindo score 3A or higher. There was no missing data in the data extraction process.

### Statistical analysis

Two reviewers (RIR and GN) performed data analysis independently. Meta-analysis of selected studies was performed using the Review Manager (RevMan) (RRID:SCR_003581) 5.3 application. Alternatively, meta-analysis of selected studies can be performed using STATA (RRID:SCR_012763). For dichotomous variables, the results were presented as odds ratio (OR) with 95% confidence interval (CI). Whereas for continuous variables, the results were presented as the mean difference with 95% CI. Heterogenicity was analyzed using the chi-squared and I ^2^ test, as appropriate. The data were analyzed using the random-effect model when I ^2^>50% and fixed-effect model when I ^2^<50%. Statistical significance was set at p<0.05. Missing data were analyzed in the outcome. For studies that provided minimum and maximum values instead of standard deviation (SD) for the mean difference analysis, estimated SD was then calculated by the formula provided by Walter and Yao (2007). ^10^ Additionally, for studies that provided 95% CI values instead of SD, SD was then calculated using the formula described in the Cochrane Handbook. ^12^

## Results

### Literature search

**Figure 1.**
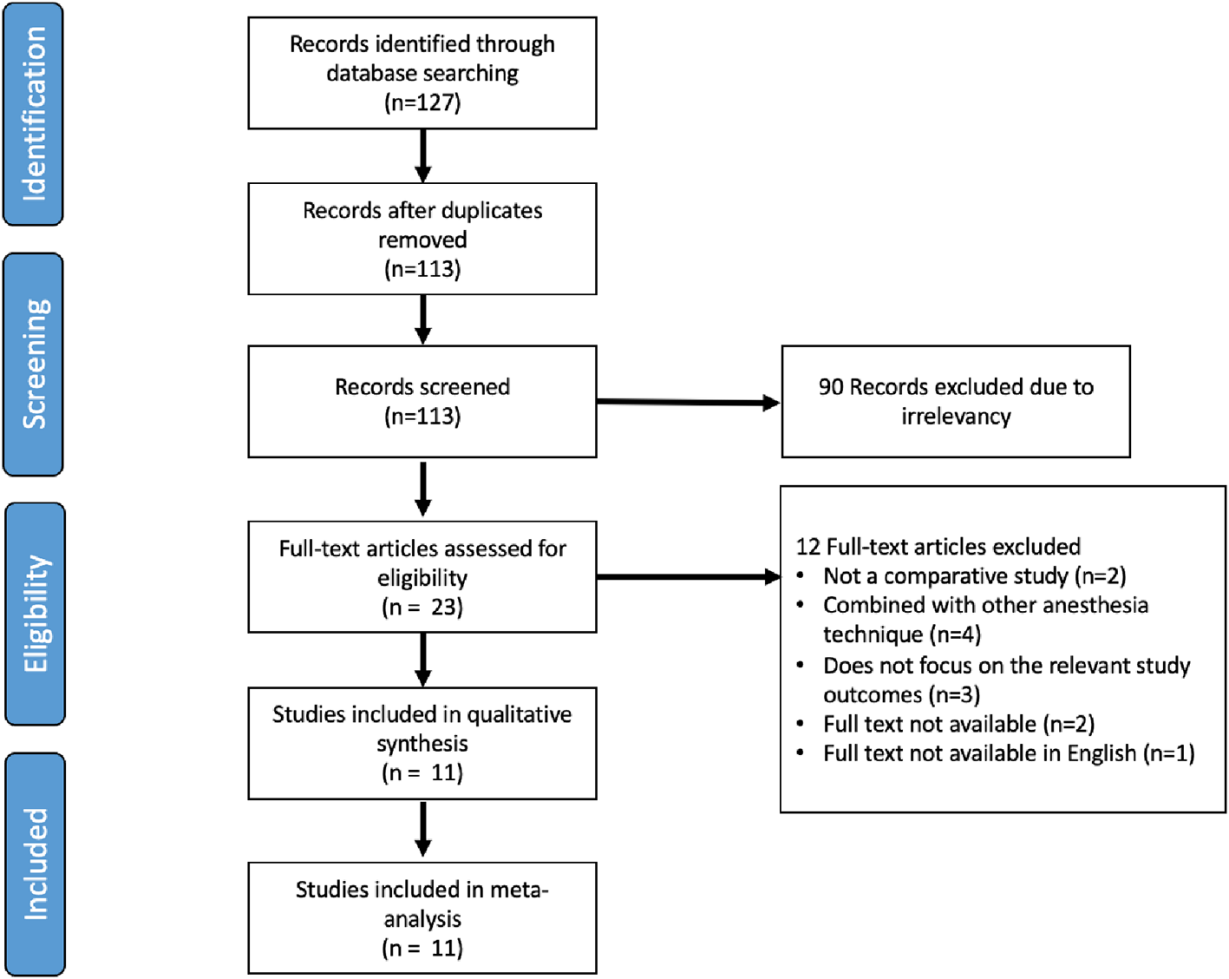
Study flow diagram.

After screening the articles and applying the inclusion and exclusion criteria, we found 127 articles from three databases. After removing duplicates, we included 113 studies. Among these, we excluded 90 studies as they were irrelevant based on their titles and abstracts. Subsequently, after assessing the full text, we included 11 studies in the final qualitative and quantitative (meta-analysis) analyses ( Figure 1).

### Study characteristics

A total of four RCTs and eight retrospective studies were assessed. The retrospective studies were assessed using Crombie's items ( Table 1) and RCTs were assessed using the Jadad Scale ( Table 2). ^12^^,^^13^ There were three grade B and four grade C retrospective studies. All the RCTs had a score of less than three. Anesthesia and PCNL methods for each study are presented in Table 3. Furthermore, study characteristics, such as number of patients, stone burden, mean age, stone free rate, follow-up period, and complication rate are presented in Table 4.

**Table 1.** Appraisal of methodological quality of the cross-sectional studies using Crombie’s items.

Author, year	Design	Data	Response rates	Representativeness	Reliable and valid measurements	Statistical significance	Statistic methods	Crombie’s score	Grading *
Gonen *et al.*(2014) ^5^	1	0.5	0	0	0.5	1	0.5	3.5	C
Karatag T *et al.* (2015) ^6^	1	1	0	0	0	1	1	4	B
Mehrabi S *et al.* (2013) ^8^	1	0.5	0	0	0	1	0.5	3	C
Cicek T *et al.* (2014) ^13^	1	1	1	0	0.5	1	0.5	5	B
Solakhan M *et al.* (2019) ^14^	1	1	0	0	0	1	1	4	B
Astram A *et al.* (2015) ^15^	1	0	0	0	0.5	1	0.5	3	C
Buldu I *et al.* (2016) ^16^	1	1	0	0	0	1	0.5	3.5	C

* Grade A (6.0-7.0), Grade B (4.0-5.5), Grade C (<4).

**Table 2.** Appraisal of methodological quality of the randomized control trials using Jadad scale.

Author, year	Randomized	Double-blind	Withdrawals	Randomization method	Double-blinding described	Score *
Nouralizadeh A *et al.* (2013) ^17^	1	0	0	0	0	1
Movasseghi G *et al.* (2014) ^18^	1	0	0	1	0	2
Shah T *et al.* (2016) ^7^	1	0	0	1	0	2
Bhattarai R *et al.* (2016) ^19^	1	0	0	0	0	1

* Score below 3 considered as poor.

**Table 3.** Anesthesia and PCNL technique in the studies.

Author	Country	Study Design	PCNL Technique				Anesthesia	
			Position	Imaging	Dilation	Fragmentation	Spinal	GA
Mehrabi S *et al.* (2013) ^8^	Iran	Retrospective study	Supine	Fluoroscopy	One-shot technique using Amplatz dilator 28F/30F	Pneumatic & shockwave lithotripter	Bupivacaine 0.5% 2–2.5 ml + 0.5 mL fentanyl at L4 & L5 intervertebral space	Midazolam, thiopental, atracurium (dose not specified)
Nouralizadeh A *et al.* (2013) ^17^	Iran	RCT	Prone	Fluoroscopy	Single-stage technique	Pneumatic lithotripter & holmium laser	Bupivacaine 0.5% 0.25 mg/kg (up to 40 mg) at L3-L4 at intervertebral space	Fentanyl 2 mg/kg, midazolam 0.03 mg/kg, propofol 2 mg/kg, atracurium 0.5 mg/kg
Cicek T *et al.* (2014) ^13^	Turkey	Retrospective study	Prone	Fluoroscopy	Amplatz dilation	Pneumatic lithotripter	Levobupivacaine 15–20 mg + midazolam 2.5 mg at L2-L3 intervertebral space	Propofol 2.5 mg/kg, 1 mg/kg fentanyl, 0.5 mg/kg atracurium intravenously
Movasseghi G *et al.* (2014) ^18^	Iran	RCT	Prone	Fluoroscopy	N/A	N/A	Bupivacaine 0.5% 15–20 mg + 0.01–0.02 mg/kg midazolam	Fentanyl 1–2 mg/kg, midazolam 0.01–0.02 mg/kg, thiopental-Na 3-5 mg/kg, atracurium 0.5 mg/kg, propofol 100 mcg/kg/min
Gonen *et al.* (2014) ^5^	Turkey	Retrospective study	Prone	Fluoroscopy	Amplatz dilation	Pneumatic Lithotripter	Bupivacaine 8–15 mg at L2-L3 intervertebral space	Propofol 2 mg/kg, fentanyl 1 mg/kg, rocuronium bromide 0.6 mg/kg
Karatag T *et al.* (2015) ^6^	Turkey	Retrospective study	Prone	Fluoroscopy	4.8Fr “all-seeing needle” microperc system	Holmium: YAG laser	Levobupivacaine 15–20 mg at L3-L4/L4-L5 intervertebral space	Thiopental 5 mg/kg, fentanyl 2 mcg/kg, atracurium 0.5 mg/kg
Astram A *et al.* (2015) ^15^	Indonesia	Retrospective study	N/A	N/A	N/A	N/A	N/A	N/A
Buldu I *et al.* (2016) ^16^	Turkey	Retrospective study	Prone	Fluoroscopy	Amplatz dilation	Pneumatic lithotripter	Bupivacaine 15–20 mg + midazolam 2 mg sedation at L3-L4 intervertebral space	Propofol 2 mg/kg, fentanyl 1 mg/kg, unspecified neuromuscular block
Shah R *et al.* (2016) ^7^	Nepal	RCT	Prone	Fluoroscopy	Serial dilators 26-30 F	Pneumatic Lithotripter	Bupivacaine 0.5 % + 0.5 ml fentanyl (25 mcg) at L3-L4 intervertebral space	Midazolam 40 mcg/kg, fentanyl 2 mcg/kg, propofol 2 mg/kg, vecuronium 0.1 mg/kg
Bhattarai R *et al.* (2016) ^19^	Nepal	RCT	N/A	N/A	N/A	N/A	0.5% bupivacaine (hyperbaric) at L3-L4 intervertebral space	Midazolam 0.04 mg/kg, fentanyl 1 mcg/kg, propofol 2.5 mg/kg & Inj. atracurium 0.5 mg/kg
Solakhan M *et al.* (2019) ^14^	India	Retrospective study	Prone	Fluoroscopy	Amplatz dilation	N/A	Bupivacaine 20 mg 0.5% + midazolam 2 mg at intervertebral space	Propofol 2 mg/kg, fentanyl 1 mg/kg, 0.5 rocuronium bromide mg/kg, midazolam 2 mg/kg

PCNL, percutaneous nephrolithotomy; RCT, randomized controlled trials; GA, general anesthesia; N/A, not applicable.

**Table 4.** Characteristics of included studies.

Author	Study design	Case		Stone size		Mean stone number		Stone free rate (%)				Complications (%)		
		Spinal	GA	Spinal	GA	Spinal	GA	Spinal	GA	Definition	Follow-up time (days)	Spinal	GA	Definition
Mehrabi S *et al.* (2013) ^8^	Retrospective Study	58	52	31.3±7.9 mm	34.2±9.8 mm	N/A	N/A	79.3	80	Stone ≤4 mm	1	15.5	11.5	N/A
Nouralizadeh A *et al.* (2013) ^17^	RCT	50	50	5.51±2.87 cm ^2^	5.56±2.95 cm ^2^	N/A	N/A	78	84	Stone ≤4 mm	1	30	40	Clavien–Dindo I-V
Cicek T *et al.* (2014) ^13^	Retrospective Study	440	564	533.9±480.94 mm ^2^	529.5±324.12 mm ^2^	N/A	N/A	73.6	73.9	Stone ≤4 mm	Immediate postoperative	18.6	20.6	Clavien–Dindo I-V
Movasseghi G *et al.* (2014) ^18^	RCT	30	29	N/A	N/A	N/A	N/A	N/A	N/A	N/A	N/A	N/A	N/A	N/A
Gonen *et al.* (2014) ^5^	Retrospective Study	20	26	558.6±297.2 mm ^2^	630.7±486.2 mm ^2^	N/A	N/A	96.2	95	Absence of residual stone	1	7.70	5	Clavien–Dindo I-V
Karatag T *et al.* (2015) ^6^	Retrospective Study	63	53	155.08±84.9 mm ^2^	151.00±75.5 mm ^2^	1.4±0.69	1.3±0.59	93.6	90.5	Absence of residual stone	1	9.40	9.3	Clavien–Dindo I-IIIa
Astram A *et al.* (2015) ^15^	Retrospective Study	540	220	36.76±17.66 mm	40.93±22.87 mm	N/A	N/A	73.0	71.4	N/A	N/A	5.18	9.5	N/A
Buldu I *et al.* (2016) ^16^	Retrospective Study	47	53	52.9±15.4 mm	50.6±24.6 mm	N/A	N/A	61.7	52.8	Absence of residual stone	N/A	19.1	13.2	Clavien–Dindo I-V
Shah R *et al.* (2016) ^7^	RCT	30	30	3.23±1.36 cm	3.75±1.27 cm	Single or multiple	Single of multiple	93.3	83.3	Absence of residual stone	1	13.3	16.7	Clavien–Dindo I-V
Bhattarai R *et al.* (2016) ^19^	RCT	30	30	27.6±5.8 mm	26.3±6.6 mm	3.8±3.1	3.4±3.6	N/A	N/A	N/A	N/A	N/A	N/A	N/A
Solakhan M *et al.* (2019) ^14^	Retrospective Study	1,085	572	635.2±304.1 mm ^2^	644.5±301.8 mm ^2^	N/A	N/A	85.1	83.4	Stone ≤4 mm	N/A	24.4	23.8	Clavien–Dindo I-V

RCT, randomized controlled trials; GA, general anesthesia; N/A, not applicable.

### Operation time

**Figure 2.**
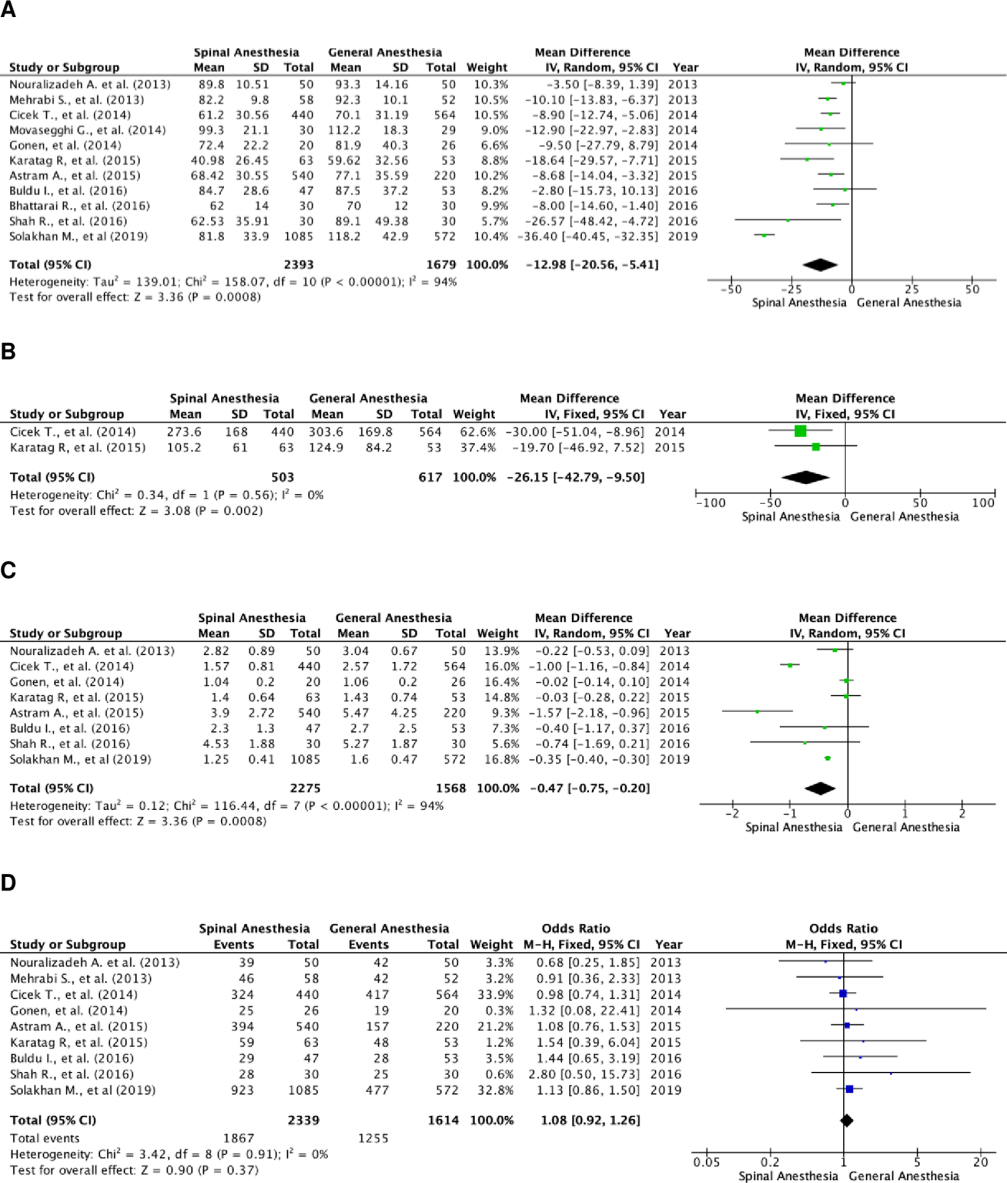
Forest plot of the study outcomes. (A) Pooled estimate of operation time using random-effect model; (B) pooled estimate of fluoroscopy time using fixed-effect model; (C) pooled estimate of length of stay using random-effect model; (D) pooled estimate of stone-free rate using fixed-effect model.

A total of 4,072 patients were quantitatively analyzed for operation time in the included studies, with 2,393 patients in the SA and 1,679 in the GA group ( Figure 2A). High heterogenicity was detected in these studies (I ^2^=94%), and therefore a random-effects model analysis was performed. Pooled data showed that SA had a significantly faster operation time, as compared to GA, using the random-effect model analysis, and the mean difference for SA *versus* GA was -12.98 minutes (95% CI, 20.56 to -5.41; p=0.0008).

### Fluoroscopy time

There were two studies that compared fluoroscopy time between the two groups ( Figure 2B). A total of 1,120 subjects were included, with 503 in the SA group and 617 in the GA group. The studies were homogenous (I ^2^=0%). The SA group had a significantly faster fluoroscopy time as compared to the GA group, with a fixed effect mean difference of -26.15 minutes (95% CI, -42.79 to -9.50; p=0.002).

### Length of stay

There were eight studies that assessed the length of hospital stay of the patients. A total of 3,843 patients were included, with 2,275 in the SA group and 1,568 in the GA group ( Figure 2C). These studies were heterogenous with I ^2^=94%. Patients in the SA group were discharged sooner as compared to those in the GA group. The result of the random-effects model was statistically significant with a mean difference of -0.47 day (95% CI, -0.75 to -0.20; p=0.0008).

### Stone-free rate

There was a total of nine studies that reported the stone-free rate of patients in both groups. There were 3,953 patients in total, and 2,339 patients belonged to the SA group and 1,614 to the GA group ( Figure 2D). Heterogenicity was not found in these studies with I ^2^=0%. Hence, a fixed-effect analysis was performed. There was no significant difference in the stone-free rate between the two groups, with a fixed-effect odds ratio of 1.08 (95% CI, 0.92 to 1.26; p=0.37).

### Overall complication rate (classified by the Clavien–Dindo Score)

**Figure 3.**
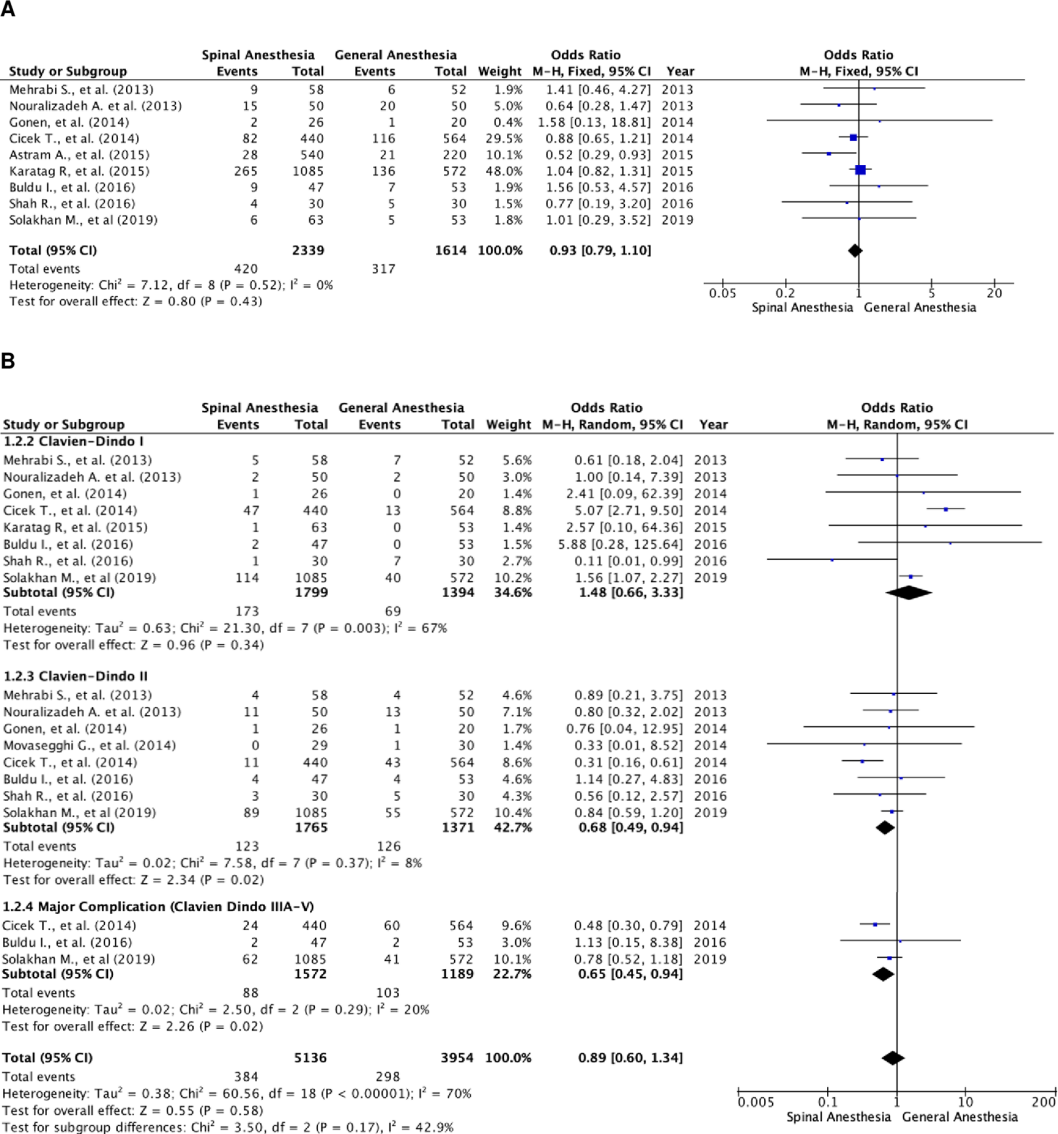
Forest plot of complication rate of the studies. (A) Pooled estimate of overall complication rate using fixed-effect model; (B) pooled estimate of every Clavien–Dindo classification complication.

There were nine studies that reported the overall complication rate ( Figure 3A). ^5^^,^^18^^,^^20^ A total of 3,953 patients were included, of which 2,339 patients were in the SA group and 1,614 patients in the GA group. The studies were homogenous (I ^2^=0%). There was no statistically significant difference in the overall complication rates between the two groups. The fixed-effect model’s odds ratio was 0.93 (95% CI, 0.79 to 1.10; p=0.43).

Further subgroup analysis was performed in relation to every Clavien–Dindo classification ( Figure 3B). There were 9,090 events of complications noted, of which 5,136 were in the SA group and 3,954 in the GA group. In these studies, one patient could experience more than one complication, resulting in a higher number of events compared to the total number of patients with complications ( Figure 3A). Heterogenicity was noted in patients with Clavien–Dindo grade I complications (I ^2^=67%) and major complication rate (I ^2^=70%). There were notably more patients with Clavien–Dindo grade II and major complications in the SA group as compared to the GA group. The result was statistically significant with a fixed-effect model odds ratio of 0.68 (95% CI, 0.49 to 0.94; p=0.02) and random-effect model odds ratio of 0.65 (95% CI, 0.45 to 0.94; p=0.29). However, there were no differences in Clavien–Dindo grade I complication rate with a random-effect model odds ratio of 1.48 (95% CI, 0.66 to 3.33; p=0.34).

### Specific postoperative complications

**Figure 4.**
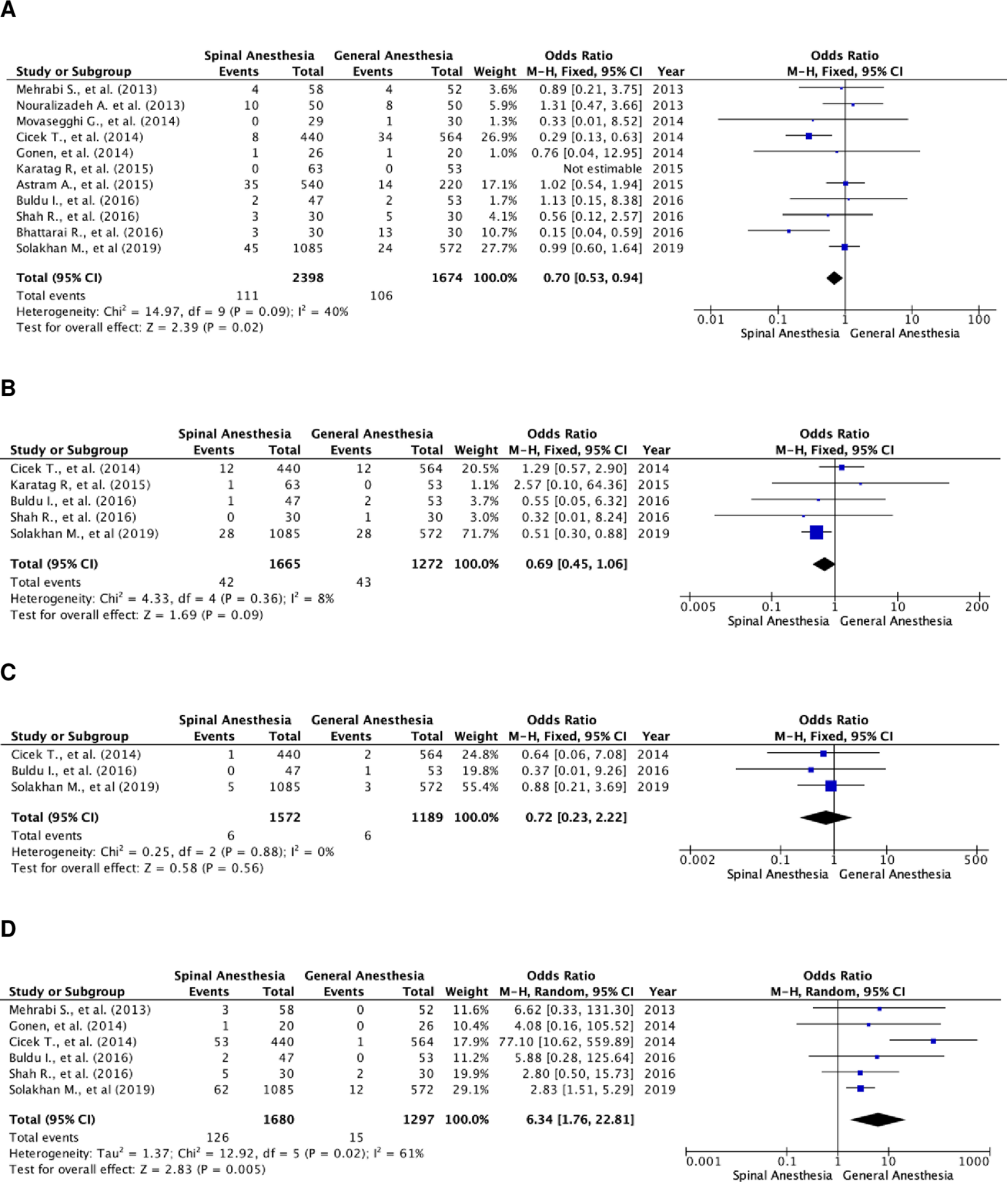
Forest plot of specific complication parameters of the studies. (A) Pooled estimate of transfusion rate using fixed-effect model; (B) pooled estimate of UTI using fixed-effect model; (C) pooled estimate of urosepsis using fixed-effect model; (D) pooled estimate of postoperative headache using random-effect model. UTI, urinary tract infection.

Further analysis of postoperative complications showed that patients in the SA group had higher transfusion rates ( Figure 4A). The odds ratio of the fixed-effects model was 0.70 (95% CI, 0.53 to 0.94; p=0.02). There were 11 studies that included transfusion rate as a parameter, with a total of 4,072 subjects, of which 2,398 were in the SA group and 1,674 in the GA group. The studies were not heterogenous (I ^2^=40%).

There was no significant difference in UTI and urosepsis between the two groups ( Figure 4B and C). Meanwhile, patients in the GA group experienced more postoperative headaches ( Figure 4D). The odds ratio of the random-effect model was 6.34 (95% CI, 1.76 to 22.81; p=0.005). There were six studies that reported postoperative headache as a complication, with a total of 2,977 patients, of which, 1,680 patients were in the SA group and 1,297 patients were in the GA group. Heterogenicity was noted with I ^2^=61%.

### Cost

Only one study discussed the cost difference between the GA and SA groups. Mehrabi *et al.*, stated that GA was notably more expensive than SA. The cost of drugs and materials were USD 5.4±3.1 and USD 23±7.3 for SA and GA, respectively. ^8^

### Pain score (visual analog score (VAS))

Only one study discussed the visual analog score (VAS) between both the groups. Karatag *et al.*, showed that there was no significant difference (p=0.365) in VAS in both groups. The VAS of the SA and GA group were 3.0±1.3 and 2.9±1.7, respectively. ^6^

### Postoperative analgesic requirement

Postoperative analgesic requirement was described in three studies. Overall, these studies stated that patients in the SA group required significantly less postoperative analgesic drugs, as compared to those in the GA group. Gonen *et al.*, found that patients administered SA (53.8±39.8 mg) require significantly less postoperative intravenous tramadol, as compared to those administered GA (111.5±46.3 mg; p<0.001). ^5^ In addition, Mehrabi *et al.*, also stated that patients in the SA group require significantly less postoperative intravenous opioid drugs (unspecified), as compared to those in the GA group on the first (SA: 7.8±2.3 mg, GA: 12.4±3.1 mg; p=0.03) and second (SA: 11.1±2.1 mg, GA: 13.2±2.1 mg; p=0.06) postoperative days. ^8^ However, the difference in opioid drug requirement on the second postoperative day was not significant. Moreover, Bhattarai *et al.*, stated that patients in the SA group (8.2±1.2 mg) require significantly less postoperative analgesic drugs (unspecified) compared to those in the GA group (14.6±2.4 mg; p=0.0001). ^19^

## Discussion

This systematic review and meta-analysis investigated the efficacy and safety profile of SA compared to GA in PCNL. The best study design to evaluate such type of study is RCT. This study included four RCTs. This study finds that SA and GA are both equally safe and effective for PCNL. Key differences are that GA resulted in fewer Clavien-Dindo grade II complications, major complications, and lower transfusion rates. SA resulted in faster operation time, fluoroscopy time, reduced length of stay, and lower postoperative analgesic requirement and cost.

PCNL is the first-line treatment for large nephrolithiasis, sized >20 mm. ^1^^,^^2^ This procedure is traditionally performed after administering GA. ^20^ GA may result in fluid absorption and electrolyte imbalance, therefore performing GA in patients with comorbidities could be challenging. For patients with chronic cardio-pulmonary conditions, such as chronic obstructive pulmonary disease or chronic heart failure, SA may be the method of choice for anesthesia. ^8^^,^^21^ Moreover, GA has potential adverse effects such as allergic drug reactions, cardiopulmonary compromise, and aspiration of gastric contents. There are several studies in which SA was performed for PCNL candidates who were critically-ill and morbidly-obese to avoid cardiorespiratory compromise during the procedure. ^9^^,^^22^^,^^23^

To date, there is no consensus concerning the best mode of anesthesia for PCNL. To address this matter, in 2015, Pu *et al.*, published a meta-analysis comparing GA to regional anesthesia (SA, epidural anesthesia, and spinal-epidural anesthesia). ^24^ To the best of our knowledge, this is the first meta-analysis directly comparing the efficacy and safety of GA and SA in patients who underwent PCNL.

In a majority of the studies in this review, PCNL was performed in the prone position ^5^^–^^7^^,^^13^^,^^14^^,^^16^^–^^18^; except for one study by Mehrabi *et al.*, in which PCNL was performed in the supine position. ^8^ PCNL performed in the prone position results in a higher stone-free rate than that performed in the supine position. In regard to the safety profile, performing PCNL in the supine position yields superior results than in the prone position. ^4^ The supine position also makes it easier for anesthesiologists to handle cardiorespiratory emergencies intraoperatively, as compared to the prone position. ^4^

The authors chose stone-free rate as the study's primary outcome to compare both the methods of anesthesia from a urologist’s perspective; the secondary outcomes are operation time, fluoroscopy time, length of stay, and complications. In this study, we found that GA is superior in terms of lower Clavien–Dindo grade >II complications, and lower transfusion rate and UTIs, whereas SA is superior in terms of shorter operation time, fluoroscopy time, length of stay, and significantly fewer cases of postoperative headache. Both methods are similar in terms of stone-free rate and overall complication rate. Therefore, surgeons can freely choose between GA or SA for PCNL without having to worry about the efficacy of the anesthesia method.

Every study included in this review reported faster operation time in the SA group as compared to the GA group. The operation and fluoroscopy time was faster in the SA group. In most studies included in this review PCNL was performed in the prone position. When administering GA for PCNL, the patient must be positioned twice. The patient initially must lie in the supine position to be intubated. Thereafter, the patient is pronated for PCNL. This two-stage nature of the procedure may attribute to a longer operation and fluoroscopy time in the GA group. ^3^

Length of hospital stay is shorter in the SA group. This may be attributed to the reduced risk of systemic complications with SA as compared to GA. The method of anesthesia can affect early postoperative recovery for patients. SA is typically performed by administering bupivacaine. A study has demonstrated sensory and motor blockade for 133.16 ± 42.21 minutes after the use of bupivacaine. ^25^ These findings correlate well with the reduced requirement of postoperative analgesics for patients in the SA group as compared to those in the GA group because SA has a lingering effect that lasts post-surgery. A study by Mehrabi *et al.*, showed that on the first postoperative day, patients in the SA group required significantly lower doses of postoperative intravenous opioid drugs, as compared to the GA group. ^8^

Overall complications did not differ between the two groups. In terms of specific complications, patients in the GA group experienced more postoperative headaches as compared to those in the SA group. In patients who were administered GA, postoperative headache, nausea, and vomiting were a common finding. ^26^ A prospective study assessing the postoperative complications of GA in oral and maxillofacial surgery reported that 41% of the subjects experienced postoperative headaches. On the contrary, SA has fewer systemic effects, resulting in less frequently reported postoperative headaches. ^26^

Bleeding is a well-known complication in PCNL. In our meta-analysis we found that patients in the SA group had a higher transfusion rate. This result contradicts previous meta-analyses, where SA is associated with a significant decrease in blood loss compared to GA in surgeries within the pelvic, abdominal, and thoracic cavities and lower extremities. ^27^

Mehrabi *et al.*, reported that GA is more expensive than SA. The cost of drugs and materials are USD 5.4 ± 3.1 (SA) and USD 23 ± 7.3 (GA). Previous studies on orthopedic surgeries have also demonstrated that SA is relatively less expensive as compared to GA. ^28^ Possible heterogeneity in this study may be caused by differences in sample size, anesthesia drug, and stone fragmentation method.

The limitation of this study is that there are few articles that have reported fluoroscopy time in both the groups. Postoperative analgesic consumption was also difficult to compare between the studies because there was no uniform term for the definition of postoperative analgesic consumption. Few studies have reported major complication rates using the Clavien–Dindo classification, including the incidence of urosepsis. There are only four RCTs included, and the rest of the studies are retrospective studies. Therefore, there could be an overestimation of the result due to selection bias. The RCTs assessed had low scores due to lack of blinding. However, double blinding is no applicable in the studies. Patient must provide consent to be included in the study and anesthesiologists performing anesthesia must be prepared for the upcoming procedure to ensure patient safety. Therefore, the anesthesia modality must be specified prior to the procedure. In this case, a third observer must be involved to assess the outcomes of the subjects without knowing what group they are in to ensure objectivity. There is a limitation in the review process, which is that there were only two reviewers of the articles in this study.

However, this study addresses the advantages and disadvantages of both GA and SA in PCNL, with an aim to provide valuable insights for surgeons to choose the most appropriate method of anesthesia for PCNL.

## Conclusions

In terms of efficacy marked by stone-free rate, there are no significant differences between GA and SA **.** GA is superior in terms of lower Clavien–Dindo grade II complications, transfusion rate, and UTI occurrence, whereas SA is superior in terms of shorter operation time, fluoroscopy time, length of stay, and a significantly lower frequency of postoperative headaches. Both methods are similar in terms of the overall complication rate. Therefore, the decision to choose between GA and SA should be based on the patient's clinical parameters and the surgical team’s preferences.

## Data availability

### Underlying data

All data underlying the results are available as part of the article and no additional source data are required.

### Reporting guidelines

Open Science Framework: PRISMA checklist for article 'General versus spinal anesthesia in percutaneous nephrolithotomy: A systematic review and meta-analysis', https://doi.org/10.17605/OSF.IO/7KR58. ^29^

Data are available under the terms of the Creative Commons Zero “No rights reserved” data waiver (CC0 1.0 Public domain dedication).
