# Supplementary material for: General versus spinal anesthesia in percutaneous nephrolithotomy: A systematic review and meta-analysis
Source: F1000Res. 2023 Nov 1;12:281. Originally published 2023 Mar 14. [Version 2] doi: 10.12688/f1000research.124704.2 (PMC11016174; doi:10.12688/f1000research.124704.2)
Supplement: Peer review history

# Reviewer report: Reviewer response for version 2

**DOI:** 10.5256/f1000research.158282.r265171

**Published:** 2024-04-13

**Authors:** Tanan Bejrananda

**Affiliations:** 1 Prince of Songkla University, Songkhla, Thailand

Overall, this manuscript provides a comprehensive review and meta-analysis of the efficacy and safety of spinal anesthesia (SA) compared to general anesthesia (GA) in percutaneous nephrolithotomy (PCNL). Here are some comments on different sections of the manuscript:

 1. **Introduction:**

    - The introduction provides a clear overview of the prevalence of nephrolithiasis and the importance of PCNL as a treatment modality.

    - It effectively sets up the need for the study by highlighting the conflicting evidence regarding the choice between GA and SA for PCNL.

 2. **Methods:**

    - The methods section is detailed and well-structured, providing clarity on the study design, inclusion criteria, and outcome measures.

    - The use of the Clavien–Dindo classification system for assessing complication rates adds rigor to the analysis.

    - The explanation of data extraction and statistical analysis methods is thorough and helps readers understand how the results were obtained.

 3. **Results:**

    - The results are presented clearly with the use of forest plots, making it easy to interpret the findings of each outcome measure.

    - The inclusion of subgroup analyses adds depth to the discussion of complication rates, providing insights into specific categories of complications.

    - The discussion of specific postoperative complications, such as transfusion rates and UTIs, adds valuable information to the overall assessment of safety.

 4. **Discussion:**

    - The discussion effectively synthesizes the findings of the study and contextualizes them within the existing literature.

    - It provides insights into the clinical implications of the results, highlighting the potential advantages and disadvantages of both anesthesia methods for PCNL.

    - The discussion of the limitations of the study, such as the heterogeneity of included studies and potential biases, demonstrates a thoughtful approach to data interpretation.

 5. **Figures and Tables:**

    - The figures and tables are well-designed and complement the text by visually summarizing key findings and study characteristics.

    - The forest plots provide a clear visual representation of the meta-analysis results, aiding in the understanding of effect sizes and confidence intervals.

 Overall, the manuscript is well-written, and the findings are presented in a structured and logical manner. The authors have conducted a thorough review and meta-analysis, providing valuable insights into the choice between GA and SA for PCNL. However, some minor revisions, such as clarifications on specific terminology and formatting improvements, could further enhance the manuscript's clarity and readability.

Are the rationale for, and objectives of, the Systematic Review clearly stated?

Yes

Is the statistical analysis and its interpretation appropriate?

Yes

Are sufficient details of the methods and analysis provided to allow replication by others?

Yes

Are the conclusions drawn adequately supported by the results presented in the review?

Yes

Reviewer Expertise:

uro-oncology, endourology, kidney transplantation

I confirm that I have read this submission and believe that I have an appropriate level of expertise to confirm that it is of an acceptable scientific standard.

## References

[1] : Successful Percutaneous Nephrolithotomy Using Thoracic Paravertebral Block as the Sole Surgical Anesthesia: Cases Report. *Journal of Health Science and Medical Research* .2021; 10.31584/jhsmr.2021788 10.31584/jhsmr.2021788

# Reviewer report: Reviewer response for version 2

**DOI:** 10.5256/f1000research.158282.r220001

**Published:** 2023-11-08

**Authors:** M. Hammad Ather

**Affiliations:** 1 Section of Urology, Department of Surgery, Aga Khan University Hospital, Karachi, Pakistan

Authors have incorporated the recommendations and the article looks more balanced now. I have no further suggestions.

Are the rationale for, and objectives of, the Systematic Review clearly stated?

No

Is the statistical analysis and its interpretation appropriate?

Yes

Are sufficient details of the methods and analysis provided to allow replication by others?

Yes

Are the conclusions drawn adequately supported by the results presented in the review?

Partly

Reviewer Expertise:

Endourology and Uro oncology

I confirm that I have read this submission and believe that I have an appropriate level of expertise to confirm that it is of an acceptable scientific standard.

# Reviewer report: Reviewer response for version 1

**DOI:** 10.5256/f1000research.136928.r171948

**Published:** 2023-07-13

**Authors:** M. Hammad Ather

**Affiliations:** 1 Section of Urology, Department of Surgery, Aga Khan University Hospital, Karachi, Pakistan

Authors have performed a meta analysis comparing spinal anesthesia with the standard GA in performing PCNL for >20mm kidney stones. They noted that there is no clear winner and both can be safely used with matching efficacy. Authors have noted that the quality of studies and limited number of RCTs rendered the conclusions rather weak. In the discussion section authors could not provide a compelling evidence of better complication rate in GA patients and shorter fluoroscopy and operative time in regional anesthesia. They also failed to identify which type is suited when, which perhaps is the most important question for the clinicians. It is also important for the future investigators to assess operator experience (both urologist and anesthetist) with regional anesthesia and also potential difficulties and monitoring of the patient undergoing prone PCNL under regional anesthesia. In a recently reported RCT on 90 patients authors noted no major difference in the two types (GA and combined spinal epidural).

Are the rationale for, and objectives of, the Systematic Review clearly stated?

No

Is the statistical analysis and its interpretation appropriate?

Yes

Are sufficient details of the methods and analysis provided to allow replication by others?

Yes

Are the conclusions drawn adequately supported by the results presented in the review?

Partly

Reviewer Expertise:

Endourology and Uro oncology

I confirm that I have read this submission and believe that I have an appropriate level of expertise to confirm that it is of an acceptable scientific standard, however I have significant reservations, as outlined above.

## References

[1] : A Randomized Control Trial to Compare Hemodynamic Parameters of Patients Undergoing Percutaneous Nephrolithotomy Under Combined Spinal-Epidural and General Anesthesia in a Tertiary Hospital. *Local Reg Anesth* .2023;16: 10.2147/LRA.S410510 41-49 10.2147/LRA.S410510 37223489 PMC10202212

# Response

**Published:** 2023-10-29

**Authors:** Rinaldo Indra Rachman

Spinal anesthesia is generally preferred than general anesthesia, as SA yields minimal systemic effect compared to GA. Using GA increases the risk of anaphylaxis because of the drug used in GA. However GA eases the procedure by controlling the breathing and renal movement of the patient.

# Reviewer report: Reviewer response for version 1

**DOI:** 10.5256/f1000research.136928.r167852

**Published:** 2023-05-02

**Authors:** Noor Buchholz

**Affiliations:** 1 U-merge Ltd. (Urology for Emerging Countries), London, UK

Some criticisms:

 Introduction: 

- You write that some studies have also shown that GA costs more than SA. In Results it is written that only one study discussed the cost difference between the GA and SA groups. That does not fit together.

Methods:

- The age and gender of the subjects in the individual studies were not specified.
- No indication of group sizes under methods.
- According to the size of the groups in Table 4, there are clear size differences in some retrospective studies between GA and spinal anesthesia. Why? Could that be a bias?
- The objective/ the target points are described in more detail in the abstract than in the actual review.
- One of the targets, the Clavien-Dindo scoring system, could possibly be briefly explained.

Results:

- Unfortunately, the results section is divided and presented in a very confusing manner. In addition, a very confusing distribution of the results across Table 4 and Figures 2, 3 and 4. The results should perhaps be presented consistently either as in Table 4 or in Figures 2, 3 and 4. Presentation better not in the text, but possibly at the end.
- Literature search and study characteristics should perhaps be listed under Methods.
- Number of studies varies again and again in varying numbers; sometimes 11, then again 12.
- The mean age of the subjects is mentioned under study characteristics, but no information was actually given in Table 4.
- In some cases only individual result points are evaluated from the individual studies. Why were these studies not excluded, contrary to statements under Literature search. And why were items, such as costs (discussed only in one study), not removed from the review?
- Homogeneity or heterogeneity is mentioned again and again among the various result points. What kind of homogeneity/heterogeneity of the study in relation to what is meant by it?
- Unfortunately, only one or three studies seem to be somewhat incomplete to provide a comprehensive overview of the pain score and the postoperative analgesic requirement.
- Unlike the other parts of the results, there are no figures or tables for the pain score and the postoperative analgesic requirement.
- With regard to the postoperative analgesic requirement, only opioid administration is mentioned. But not from possible other painkillers, which could falsify the statement.

Are the rationale for, and objectives of, the Systematic Review clearly stated?

Yes

Is the statistical analysis and its interpretation appropriate?

Not applicable

Are sufficient details of the methods and analysis provided to allow replication by others?

No

Are the conclusions drawn adequately supported by the results presented in the review?

Partly

Reviewer Expertise:

NA

I confirm that I have read this submission and believe that I have an appropriate level of expertise to state that I do not consider it to be of an acceptable scientific standard, for reasons outlined above.

# Response

**Published:** 2023-10-29

**Authors:** Rinaldo Indra Rachman

**Introduction:**

**Reviewer's comment**: You write that some studies have also shown that GA costs more than SA. In Results it is written that only one study discussed the cost difference between the GA and SA groups. That does not fit together.

**Author's response:**  Cost and Visual analogue score was an additional important finding of this study. However forest plot was unable to be generated because only one study addressed each of this matter. Therefore cost and visual analogue score is better suited to be included in the discussion rather than result section (already revised)

**Methods:**

**Reviewer's comment:** The age and gender of the subjects in the individual studies were not specified.

**Author's response: ** Has been added to table 4

**Reviewer's comment:** According to the size of the groups in Table 4, there are clear size differences in some retrospective studies between GA and spinal anesthesia. Why? Could that be a bias?

**Author's response:** In table 4, the study by Astram, et al included 540 patients with spinal anestesia and 220 patients with general anesthesia. There are more than twice patients in general anesthesia group because this study included all patients who underwent PCNL during 2000 - 2011 period. The same gues for the study by Cicek et al which divided samples into two groups GA (n=564) and SA (n=440). This study included all patients who underwent PCNL between 2004-2011. Both studies mentioned the decision of using GA or SA is based on each patient's characteristics, however they did not specify the characteristics. The difference of sample size between the two group may result in bias

**Reviewer's comment:** The objective/ the target points are described in more detail in the abstract than in the actual review.

**Author's response:** The objective of the study is now clearly mentioned in the methods section

**Reviewer's comment:** One of the targets, the Clavien-Dindo scoring system, could possibly be briefly explained

**Author's response:** has been added to the methods section

**Results:**

**Reviewer's comment:** Unfortunately, the results section is divided and presented in a very confusing manner. In addition, a very confusing distribution of the results across Table 4 and Figures 2, 3 and 4. The results should perhaps be presented consistently either as in Table 4 or in Figures 2, 3 and 4. Presentation better not in the text, but possibly at the end.

**Author's response:** All tables and figures in the study has it's own purpose. Table 4 serves to describe the baseline characteristics of the studies, wherease Figure 2-4 serves to explain the finding of the Meta-Analysis

**Reviewer's comment: **Literature search and study characteristics should perhaps be listed under Methods

**Author's response: **literature search has been listed under methods. Study characteristics is listed under results, as this is the finding of this article

**Reviewer's comment:**Number of studies varies again and again in varying numbers; sometimes 11, then again 12

**Author's response:** the correct number of study is 11

**Reviewer's comment: **The mean age of the subjects is mentioned under study characteristics, but no information was actually given in Table 4

**Author's response:** mean age of the subjects has been added to table 4

**Reviewer's comment:** In some cases only individual result points are evaluated from the individual studies. Why were these studies not excluded, contrary to statements under Literature search. And why were items, such as costs (discussed only in one study), not removed from the review?

**Author's response:** Cost and Visual analogue score was an additional important finding of this study. However forest plot was unable to be generated because only one study addressed each of this matter. Therefore cost and visual analogue score is better suited to be included in the discussion rather than result section (already revised)

**Reviewer's comment: ** Homogeneity or heterogeneity is mentioned again and again among the various result points. What kind of homogeneity/heterogeneity of the study in relation to what is meant by it?

**Author's response: **Homogeinity and heterogeinity was mention to address the appropriate analysis using random-effect model for variables with I2 >50. Whereas variables with I2<50 was analyzed using fixed-effect model

**Reviewer's comment:** Unfortunately, only one or three studies seem to be somewhat incomplete to provide a comprehensive overview of the pain score and the postoperative analgesic requirement.

**Reviewer's comment:** Unlike the other parts of the results, there are no figures or tables for the pain score and the postoperative analgesic requirement

**Author's response: **Visual analogue score was an additional important finding of this study. However forest plot was unable to be generated because only one study addressed each of this matter. Therefore cost and visual analogue score is better suited to be included in the discussion rather than result section (already revised)

**Reviewer's comment: **With regard to the postoperative analgesic requirement, only opioid administration is mentioned. But not from possible other painkillers, which could falsify the statement

**Author's response: **Bhattarai, et al gives the information of 2 drugs used, which are paracetamol 1 gram & morphine injection. Mehrabi et al only specified the need for opioid drugs postoperatively without mentioning other analgesics. Gonen et al stated that they chose tramadol to be given intravenously for postoperative pain management. This is perhaps because of opioid is a marker for significant
